# CBD-oil as a potential solution in case of severe tamoxifen-related side effects

**DOI:** 10.1038/s41523-023-00570-x

**Published:** 2023-08-05

**Authors:** Sanne M. Buijs, C. Louwrens Braal, Stefan A. J. Buck, Noud F. van Maanen, Lonneke M. van der Meijden-Erkelens, Heleen A. Kuijper-Tissot van Patot, Esther Oomen-de Hoop, Lotte Saes, Sophia J. van den Boogerd, Liesbeth E. M. Struik, Quirine C. van Rossum-Schornagel, Ron H. J. Mathijssen, Stijn L. W. Koolen, Agnes Jager

**Affiliations:** 1 Department of Medical Oncology, Erasmus MC Cancer Institute, Rotterdam, The Netherlands; 2 Clinical Cannabis Care Pharmacy, Breukelen, the Netherlands; 3 Department of Medical Oncology, Alexander Monro Hospital, Bilthoven, The Netherlands; 4 Department of Internal Medicine, Ikazia Hospital, Rotterdam, The Netherlands; 5 Department of Internal Medicine, Franciscus Gasthuis & Vlietland, Schiedam, The Netherlands; 6 Department of Hospital Pharmacy, Erasmus University Medical Center, Rotterdam, The Netherlands

**Keywords:** Breast cancer, Pharmacokinetics

## Abstract

Tamoxifen may lead to bothersome side effects contributing to non-compliance and decreased quality of life. Patients searching for relief are increasingly turning to cannabinoids such as CBD-oil. However, CBD-oil might affect tamoxifen pharmacokinetics (PK) through CYP2D6 inhibition. The aims of this open-label, single-arm study were (1) to determine the PK profile of tamoxifen when using CBD-oil, and (2) to subsequently investigate whether CBD-oil has a beneficial influence on side effects. Study patients had to have steady-state endoxifen concentrations ≥16 nM (conservative threshold). PK sampling and side effect assessment was done at initiation of CBD-oil and 28 days thereafter. Bio-equivalence could be concluded if the 90% confidence interval (CI) for the difference in endoxifen AUC fell within the [−20%; +25%] interval. The effect of CBD-oil on side effects was evaluated using the FACT-ES questionnaire. Endoxifen AUC decreased after CBD-oil by 12.6% (*n* = 15, 90% CI −18.7%, −6.1%) but remained within bio-equivalence boundaries. The endocrine sub-scale of the FACT-ES improved clinically relevant with 6.7 points (*n* = 26, *p* < 0.001) and health-related quality of life improved with 4.7 points after using CBD (95% CI + 1.8, +7.6). We conclude that CBD-oil, if of good quality and with a dosage below 50 mg, does not have to be discouraged in patients using it for tamoxifen-related side effects. Clinical trial registration: International Clinical Trial Registry Platform (NL8786; https://www.who.int/clinical-trials-registry-platform).

## Introduction

Tamoxifen is effective in the treatment of estrogen-receptor positive breast cancer^1,2^ and is recommended for two to three years for postmenopausal patients and up to ten years for premenopausal patients^3^. Unfortunately, tamoxifen can lead to bothersome side effects such as hot flashes, arthralgia, insomnia, and mood alterations. Forty percent of patients eventually discontinue tamoxifen therapy early, mainly due to side effects^4–6^.

Breast cancer patients searching for relief from side effects are increasingly turning to cannabinoids (CBs). CBs are a group of compounds found in the *Cannabis sativa* plant but interestingly, the human body also produces endocannabinoids^7^. One of the most active phytocannabinoids produced by Cannabis sativa is the non-psychoactive substance cannabidiol (CBD)^8,9^. CBD acts as a negative allosteric modulator of the cannabinoid receptor 1 (CB1), mostly present in the central nervous system, and as an inverse agonist of the cannabinoid receptor 2 (CB2), mostly present in the immune system. CBD can also modulate other receptors such as: opioid, dopamine, melatonin, serotonin and acetylcholine receptors^7,10^. In the United States, more than 20% of breast cancer patients used CBs during their endocrine therapy hoping to reduce side effects^11^. A recent meta-analysis suggested a small improvement in pain and quality of sleep after CBD use^12^. Most recently, a RCT did not find any symptom relief after CBD compared to placebo in an advanced cancer population^13^. Whether CBD improves tamoxifen-related side effects in breast cancer patients has never been investigated.

Tamoxifen is a prodrug metabolised mostly by the cytochrome P450 (CYP) enzyme CYP2D6 in its main and most active metabolite endoxifen^14^. Several retrospective studies have shown an exposure-response relationship of endoxifen, which led to the suggestion of efficacy thresholds varying from 9–16 nM^15–17^. Due to the complex metabolism, tamoxifen is prone to drug-drug or drug-herb interactions^18,19^. CBD might also affect tamoxifen pharmacokinetics since it is known to be a potential inhibitor of CYP2D6^20–22^. Recently, a case report about a woman treated with tamoxifen for primary breast cancer showed lower endoxifen levels when tamoxifen was combined with CBD compared to tamoxifen monotherapy^23^. However, the bioavailability of sublingual CBD in the highest over-the-counter dosage should theoretically not be sufficient for significant CYP2D6 inhibition^24^.

If CBD can diminish tamoxifen-related side effects without negative impact on tamoxifen pharmacokinetics, this could be a solution for the high frequency of tamoxifen discontinuation. Therefore, the aims of our study were (1) to determine the pharmacokinetic interaction between CBD-oil and endoxifen and (2) to investigate whether there is a beneficial effect of CBD-oil on tamoxifen-related side-effects and health-related quality of life (HR-QOL) in primary breast cancer patients.

## Results

### Patient characteristics

In total, 35 patients were enrolled in the study. Four patients were excluded before the start of the study due to voluntary withdrawal (*N* = 2), disease progression (*N* = 1), and an endoxifen level <16 nM despite dose escalation (*N* = 1). Five other patients were excluded during the study due to protocol violation (*N* = 3), personal circumstances (*N* = 1), and poor venous access for blood withdrawal (*N* = 1). There were 26 evaluable patients whereof 15 for the primary pharmacokinetic endpoint. Patient characteristics can be found in Table 1.

**Table 1:** Baseline characteristics.

*N* = 26	*N* (%) or median [IQR]	
Age	49.5	[46.8–54]
BMI (kg/m^2^)	26.1	[23.8–30.9]
Biochemistry		
ALT (U/L)	18.5	[14.0–24.5]
Creatinine (µmol/L)	69	[64.8–75]
Hemoglobin (mmol/L)	8.0	[7.8–8.4]
Leucocytes (×10^9^/L)	5.8	[4.9–7.0]
Thrombocytes (×10^9^/L)	236.5	[193.0–294.5]
Duration of adjuvant tamoxifen use (months)	13	[5–24]
WHO Performance Score		
0	16	61.5%
1	10	38.5%
Local treatment		
Lumpectomy + radiotherapy	13	50%
Mastectomy only	4	15.4%
Mastectomy + radiotherapy	9	34.6%
(Neo)adjuvant chemotherapy		
Yes	20	76.9%
No	6	23.1%
(Neo)adjuvant anti-Her2Neu therapy		
Yes	1	3.8%
No	25	96.2%
Tamoxifen dose		
20 mg	18	69.2%
30 mg	4	15.4%
40 mg	4	15.4%
CYP2D6 phenotype		
Intermediate metabolizer (IM)	13	50%
Extensive metabolizer (EM)	12	46.2%
Ultrarapid metabolizer (UM)	1	3.8%

*N* number, *IQR* interquartile range.

### Pharmacokinetics

Table 2 shows the main pharmacokinetic parameters of tamoxifen and endoxifen during tamoxifen monotherapy as compared to tamoxifen combined with CBD-oil. For endoxifen, the AUC_0–24h_ significantly decreased by 12.6% (90% CI −18.7%, −6.1%) when CBD-oil was used in addition to tamoxifen. However, the 90% CI was within the limits of bio-equivalence. Also, the C_min_ and C_max_ of endoxifen decreased significantly when using CBD-oil next to tamoxifen. There were no significant differences in tamoxifen AUC_0–24h_, C_min_ and C_max_ between tamoxifen monotherapy or tamoxifen combined with CBD-oil. Moreover, the 90% CIs of all parameters were within bio-equivalence boundaries.

**Table 2:** Tamoxifen pharmacokinetics with or without CBD.

Pharmacokinetic parameters (*N* = 15)	Tamoxifen monotherapy^a^	Tamoxifen + CBD^a^	Relative difference (%) with vs without CBD (90% CI)
Tamoxifen			
AUC_0–24h_ (nmol ∙ h ∙ L^−1^)	7100 (669)	6900 (622)	−2.8% (−7.7, +2.4%)
C_min_	272 (67)	252 (64)	−7.2% (−14.1, +0.4%)
C_max_	397(69)	392 (61)	−1.2% (−8.2, +6.4%)
Endoxifen			
AUC_0–24h_ (nmol ∙ h ∙ L^−1^)	621 (22)	542 (18)	−12.6% (−18.7, −6.1%)
C_min_	28 (22)	23 (20)	−18.2% (−23.4, −12.7%)
C_max_	33 (20)	27 (17)	−16.3% (−20.7, −11.7%)

*AUC* area under the curve, *C*_*min*_ minimum plasma concentration, *C*_*max*_ maximum plasma concentration.

^a^Geometric mean (coefficient of variation %).

The AUCs of tamoxifen and endoxifen were also analyzed for the intermediate (IM) and extensive (EM) CYP2D6 phenotype patients separately (see Supplementary Table 1). In patients with an IM CYP2D6 phenotype, the AUC of tamoxifen and endoxifen decreased significantly when using CBD-oil, while in patients with an EM CYP2D6 metabolism, the AUC of both tamoxifen and endoxifen remained comparable with the 90% CIs within the bio-equivalence boundaries. There was a significant difference between ∆AUC_PK2-PK1_ of endoxifen in IM metabolizers compared with EM metabolizers (*p* = 0.004, independent samples *t*-test). To further study this difference, the C_min_ of tamoxifen and endoxifen was determined for IM and EM CYP2D6 phenotypes separately in the total study group (*N* = 25, one patient missing due to logistic reasons). Tamoxifen C_min_ was comparable in both IM and EM CYP2D6 phenotypes. Endoxifen C_min_ decreased in a comparable range in both groups and there was no statistical difference between ∆Cmin_PK2-PK1_ of endoxifen in IM metabolizers compared with EM metabolizers (*p* = 0.48, independent samples *t*-test).

### FACT-ES scores

**Fig. 1:**
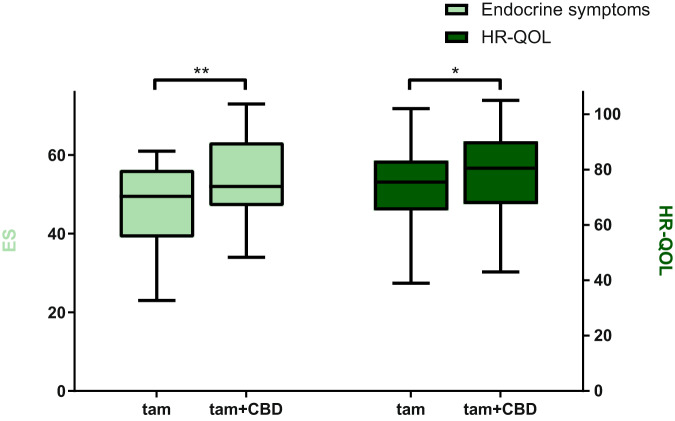
FACT-ES scores with tamoxifen monotherapy and after 4 weeks of CBD use. ES; endocrine symptoms (0–76), HR-QOL; health-related quality of life (0–108) *significant **significant and clinical relevant *one-sided paired sample *t*-test **one-sided wilcoxon signed rank test TAM mono: ES median: 49.5, IQR: 39–56, min-max: 23–61. HR-QOL median: 75.5, IQR: 65–73, min-max: 39–102 TAM + CBD: ES median: 52.0, IQR: 47–63, min-max: 34–73. HR-QOL median: 80.5, IQR: 68–90, min-max: 43–105.

In Fig. 1 a visual representation of the effect of CBD-oil on ES and HR-QOL is shown. There was a clinical relevant (≥5 points, i.e., >0.5 of SD of ES on baseline) and significant improvement in ES after four weeks of using CBD next to tamoxifen. The HR-QOL showed a significant improvement as well, but this change was not clinical relevant (<8 points, i.e., <0.5 of SD of HR-QOL on baseline). Means, confidence intervals an *p*-values can be found in Table 3.

**Table 3:** FACT-ES scores with tamoxifen monotherapy and after 4 weeks of CBD use.

FACT-ES scores (*N* = 26)	Mean without CBD	Mean after 4 weeks of CBD	Difference before and after CBD (95% CI)	*P* value (1-sided)
Health-related QOL	74.3	79.0	+4.7^a^ (+1.8, +7.6)	0.001^b^
Endocrine symptoms	47.1	53.8	+6.7^a^ (NA)	<0.001^c^

^a^Clinically relevant improvement when >0.5 of baseline SD, HR-QOL baseline SD 15.8, ES baseline SD 10.4.

^b^Paired samples *t*-test.

^c^Wilcoxon signed rank test.

To rule out the effect of the (slight) decrease in endoxifen levels on tamoxifen-related side effects instead of the effect of using CBD-oil, a Spearman correlation test was performed between the difference in endoxifen C_min_ and the improvement in ES score. No significant correlation was found (*r* = 0.19, *p* = 0.37, *N* = 25).

Joint pain improved most frequently of all separate items of the endocrine subscale of the FACT-ES (*n* = 16; 62%). Also, hot flashes, cold sweats, night sweats and insomnia (*n* = 15; 58%) and bloated feeling (*n* = 13; 50%) were items that improved frequently (i.e. in at least half of the patients). Percentage of improvement of all side effects assessed with the FACT-ES questionnaire can be found in Table 4. Improvement is seen as at least one point improvement on the 5-point Likert scale after four weeks of CBD.

**Table 4:** Side effects assessed with FACT-ES questionnaire.

Side effect	Patients with side effect on baseline (*n*)	Patients in which side effect improved (*n*)	% improved (from total patient group *n* = 26)	% improved (from patients with side effect)
Hot flashes	26	15	58%	58%
Joint pain	25	16	62%	64%
Insomnia	25	15	58%	60%
Cold sweats	24	15	58%	63%
Night sweats	23	15	58%	65%
Mood swings	21	10	38%	48%
Irritable feeling	21	10	38%	48%
Vaginal discharge	19	7	27%	37%
Vaginal dryness	19	8	31%	42%
Lost interest in sex^a^	18	5	19%	28%
Weight gain	18	7	27%	39%
Bloated feeling	18	13	50%	72%
Headache	17	8	31%	47%
Lightheaded/dizziness	16	9	35%	56%
Pain/discomfort with intercourse^a^	15	6	23%	40%
Breast sensitivity/tenderness^a^	13	9	34%	69%
Vaginal itching/irritation^a^	9	4	15%	44%
Diarrhea	6	4	15%	67%
Vomiting	3	3	12%	100%
Vaginal bleeding or spotting	2	1	4%	50%

^a^For this item some patients were missing, vaginal itching *n* = 1, pain/discomfort with intercourse *n* = 5, lost interest in sex *n* = 1, breast sensitivity/tenderness *n* = 2.

### CTCAE-toxicity

Side effects with tamoxifen monotherapy and after four weeks of tamoxifen and CBD-oil concomitantly can be found in Table 5. Hot flashes and arthralgia improved with at least one grade in six out of 25 patients (24%) and insomnia improved with one grade in 11 out of 26 patients (42%). This is in line with the trend seen in improvement in separate endocrine subscale items.

**Table 5:** CTCAE toxicity of tamoxifen with and without concomitant CBD.

	Tamoxifen-related side with tamoxifen only (*N* = 26)		Tamoxifen-related side effects with tamoxifen and CBD (*N* = 26)	
*N* (%)	Grade 1	Grade 2	Grade 1	Grade 2
Insomnia	16 (62%)	10 (38%)	19 (73%)	3 (12%)
Hot flashes	20 (77%)	5 (19%)	22 (85%)	1 (4%)
Arthralgia	15 (58%)	10 (38%)	21 (81%)	3 (12%)
Mood alterations	19 (73%)	1 (4%)	17 (65%)	1 (4%)
Muscle cramp	7 (27%)	1 (4%)	8 (31%)	–
Fatigue	3 (12%)	3 (12%)	8 (31%)	1 (4%)
Headache	4 (15%)	–	4 (15%)	–
Vaginal dryness	2 (8%)	1 (4%)	1 (4%)	–
Amnesia	4 (15%)	–	2 (8%)	–
Weight gain	2 (8%)	–	1 (4%)	–
Dry mouth	–	–	3 (12%)	
Nausea			2 (8%)	

Toxicity is shown when it occurred in more than one patient.

Ten out of 26 patients (38%) experienced some kind of CBD-oil-related toxicity. Most frequented mentioned side effects were fatigue (*n* = 3, 12%) and dry mouth (*n* = 3, 12%). All side effects were grade 1. None of the patients quit CBD-oil because of side effects. Sixty-nine percent of patients wished to continue CBD-oil after the study was finished.

## Discussion

Although the combination of CBD-oil and tamoxifen lead to a significant decrease in endoxifen plasma concentrations, the decrease remained within bio-equivalence boundaries and is not considered clinically relevant. Furthermore, CBD-oil seems to improve tamoxifen-related side effects as measured by an endocrine symptoms quality-of-life questionnaire (FACT-ES) while CBD-oil itself has only mild side effects. Thus, in case of bothersome tamoxifen-related side effects CBD addition may reduce side effects and hopefully lower the high tamoxifen discontinuation rate. However, since this was an open-label, single arm study, it is not known how much of the improvement is due to a placebo effect. HR-QOL improved significantly but this improvement was too little to be clinically relevant. Since CBD-oil was used for only four weeks this may have been too short to achieve a clinical relevant improvement in HR-QOL.

In patients with an IM CYP2D6 phenotype the decrease in endoxifen AUC seemed more pronounced than in patients with an EM CYP2D6 phenotype. Because of the additional patients recruited for side effect analysis extra information about C_min_ concentrations could be obtained. With regard to C_min_ endoxifen concentrations, there was no significant difference between IM and EM subgroups. Although C_min_ is known to be a less robust pharmacokinetic parameter than AUC, this analysis, done with a much larger subgroup, makes a difference in CBD effects between CYP2D6 phenotypes less probable.

In our study mean scores of ES and HR-QOL were 47 and 74 points, respectively. ES scores were much lower than found in previous studies where ES were assessed in large groups of unselected patients using adjuvant tamoxifen (ES: 59–62 points; HR-QOL 79–83 points)^25–27^. This is as expected, since, in contrast to our study, these populations were not selected for tamoxifen-related complaints. After four weeks of CBD, HR-QOL scores improved to an average of 79 points. While the improvement of ES scores was clinically relevant they remained below population average (54 points). Possibly, a longer period of CBD treatment can further improve ES scores.

Earlier studies suggested effects of CBD-oil on sleep and pain which was confirmed in our study^12^. Insomnia improved the most (i.e., in 42% of patients) and also, hot flashes and arthralgia improved in 24% of patients. But, a placebo effect cannot be ruled out. The most compelling study to date that placebo effect may play an important role in symptom relief from CBD oil comes from the recently published study by Hardy et al. In a randomized study they showed beneficial effect of two weeks CBD use for symptom relief in an advanced cancer population^13^. However, this effect was not statistically significantly better than placebo, suggesting an important role for a placebo effect^13^. Although a placebo-controlled randomized study is the ultimate form of ruling out a placebo effect, some nuance should be made here. The questionnaire used had a large overlap with the well-known complaints associated with CBD use. Symptoms that were assessed in this study were pain, tiredness, drowsiness, nausea, lack of appetite, shortness of breath, depression, anxiety and wellbeing. Almost half of these symptoms (tiredness, drowsiness, nausea and lack of appetite) are known side effects of CBD^28,29^. Besides, this assessment scale is hardly overlapping with any of the endocrine side effects of tamoxifen. This, in combination with the very high dose of CDB used (eight-times higher than in our study), makes it difficult to make a definitive statement about the effect of CBD-oil in case of tamoxifen-related side effects. Overall, our findings of CBD use for reducing tamoxifen-related side effects in a well-defined breast cancer patient population are promising, but certainly need further investigation in a placebo-controlled study to demonstrate the real added value of CBD-oil.

Although it is known that CBD can interact with several G-protein coupled receptors such as CB1-, CB2-, opioid-, melatonin-, acetylcholine-, serotonin- and dopamine-receptors, it is not understood how this interplay of agonism and antagonism of receptors might lead to, for example, alleviation of pain or improvement of sleep^7^. However, CB1 is highly expressed in areas in the brain related to, among others, pain, anxiety, sensory and visceral perception, motor coordination and endocrine functions^10^. We hypothesize that this might be one of the reasons that CB1 receptor activation by CBD could lead to less hot flashes and a decrease in arthralgia. Also, it is presumable that activation of other receptors as opioid- and melatonin-receptors could lead to a decrease in pain and an improvement in sleep, subsequently. This is most probably a separate mechanism not related to tamoxifen but which can coincidentally improve side effects that occur with tamoxifen. However, it is not ruled out that an interaction between the estrogen receptor and receptors where CBD engages occurs, for example in the brain where both are present.

Our study has several strengths. It is the first study investigating the pharmacokinetic interaction between tamoxifen and CBD-oil leading to highly requested knowledge for many breast cancer patients. Pharmaceutical grade CBD-oil without THC (i.e., THC < 0.05%) was used next to tamoxifen for four weeks in the highest over-the-counter dose (i.e., ≈50 mg), also securing the safety of lower CBD-oil doses. Because patients were their own control and AUCs of tamoxifen and endoxifen were measured, this led to a robust answer to this pharmacokinetic question. However, it remains unclear if higher doses of CBD, non-pharmaceutical grade CBD or other formulations of CBD are equally safe. Also, this is the first study investigating the effect of CBD-oil on tamoxifen-related side effects using the validated and reliable FACT-ES questionnaire next to CTCAE toxicity grading. A limitation of the study is the lack of a control arm when it comes to the research question about the effect of CBD use on side effects reduction. However, this is no issue for our primary, pharmacological research question. Finally, if subsequent studies confirm our results, CBD use will unfortunately not be applicable worldwide. CBD-oil is seen as a supplement in the Netherlands and many other European countries, but is on the list of prohibited narcotics in several other countries.

In conclusion, endoxifen levels remained within bio-equivalence boundaries when CBD-oil was used in combination with tamoxifen. Therefore, sublingual CBD-oil, if of good quality and not higher than the highest over-the-counter dose (<50 mg per day), does not have to be discouraged in patients using it as complementary medication. In addition, the use of CBD-oil in this single arm study resulted in a promising improvement in endocrine symptoms and quality of life, but the real effect of CBD-oil has yet to be proven in a placebo-controlled study that is currently being set up.

## Methods

This pharmacokinetic open-label single-arm, one-way cross-over study was performed at the Erasmus MC Cancer Institute in Rotterdam, The Netherlands, between November 2020 and September 2022. The study protocol was written conform the Declaration of Helsinki, approved by the Erasmus MC Medical Ethics Committee and registered at the International Clinical Trial Registry Platform (NL8786; https://www.who.int/clinical-trials-registry-platform).

### Patients

We included patients who were treated with adjuvant tamoxifen for at least three months. Steady-state endoxifen plasma concentrations had to be ≥16 nM. Furthermore, patients had to experience at least one of the following tamoxifen related side-effects, scored using US National Cancer Institute’s Common Terminology Criteria for Adverse Events version 5 (CTCAEv5): hot flashes grade ≥2, arthralgia grade ≥2, mood alterations grade ≥2 or insomnia grade ≥1. Patients were excluded if they had used CBs within three months before inclusion or if they used strong CYP3A4, CYP2D6, UDP-glucuronosyltransferase or P-glycoprotein inhibitors or inducers. All included patients gave written informed consent.

### Study design

The study started with continuation of tamoxifen monotherapy for 7 days. Patients were ordered to take their tamoxifen at 9 AM. Patients were then hospitalized for 24-h pharmacokinetic blood sampling of tamoxifen and endoxifen. Afterwards, patients started with 5 drops 10% CBD-oil sublingually three times daily for four weeks (i.e., ≈50 mg CBD per day, the highest over-the-counter dose) concomitantly to their tamoxifen treatment. The pharmaceutical grade CBD-oil was manufactured by a Dutch Pharmacy (Clinical Cannabis Care, Breukelen, the Netherlands, article number 16779517). After four weeks of concomitant CBD and tamoxifen, patients were again hospitalized for pharmacokinetic blood sampling of tamoxifen and endoxifen. Also, before and after start of CBD side effects were assessed and laboratory analysis (blood count, kidney- and liver function) was performed. Patients were asked to fill in a patient diary to verify patients’ compliance.

After the intended 15 patients completed the study protocol, the study was amended to include 11 more patients. With this amendment we were able to investigate whether CBD-oil could have a beneficial effect on tamoxifen-related side effects. Hospitalization for pharmacokinetic blood sampling was not required for these patients. A single endoxifen trough concentration (Cmin) was measured after four weeks of CBD-oil. Other design aspects of the extension part were identical to the main study.

### Pharmacokinetic and pharmacogenetic analysis

Blood samples for determination of tamoxifen and endoxifen pharmacokinetics were obtained at 13 predefined time points (*t* = 0 (before tamoxifen intake); and 0.5 h; 1 h; 1.5 h; 2 h; 2.5 h; 3 h; 3.5 h; 4 h; 6 h; 8 h; 10 h and 24 h after tamoxifen intake). Single measurements of plasma concentrations were performed on a validated liquid chromatography with a tandem mass spectrometry method (UP-LC-MS/MS)^30^. Using Phoenix WinNonLin version 8.3 the following pharmacokinetic parameters were determined or calculated: area under the plasma concentration time curves (AUC), Cmin and maximum observed plasma concentration (Cmax) of tamoxifen and endoxifen.

*CYP2D6* genotyping was performed on germline DNA using the Infiniti test (Autogenomics; Carlsblad, CA, USA) and the Quantstudio test (ThermoFisher Scientific; Waltham, MA, USA). Blood samples were assayed on the follow genetic variants: *2–10, *12, *13, *14, *17, *29, and *41.

### Quality of life and side effect

Side effects of tamoxifen and CBD-oil were assessed using CTCAEv5. To evaluate the effect of CBD-oil on tamoxifen-related side-effects and HR-QOL in more detail, patients were asked to fill in the Functional Assessment of Cancer Therapy – Endocrine Symptoms (FACT-ES) questionnaire before and four weeks after start of CBD^31^. The FACT-ES is a validated questionnaire of in total 46 questions and consists of physical, social, emotional and functional wellbeing subscales (together measuring the health-related quality of life) and an additional endocrine subscale measuring side effects of endocrine treatments give in breast cancer patients. The different endocrine subscale items and additional information about the questionnaire can be found in Supplementary Table 2.

### Statistical analysis

The primary endpoint of the study was the AUC of endoxifen. Bio-equivalence of tamoxifen with and without CBD-oil could be concluded according to Food and Drug Administration (FDA) guidelines, which suggest that the 90% confidence interval (CI) of the ratio of geometric means of the AUC should be within 0.80–1.25^32^. Given a two-sided α of 5%, 80% power, assuming a standard deviation of the difference of 20% and a true ratio of AUC geometric means of 1.0 a sample size of 15 patients was required. The AUC of endoxifen as well as all other pharmacokinetic endpoints were analyzed by means of a paired t-test on log-transformed data.

As a secondary endpoint, the ES scores of the FACT-ES questionnaire before start of CBD-oil and four weeks after start of CBD-oil were compared. Change scores of more than 0.5 of the baseline SD are considered a clinically relevant change and seen as more than a moderate effect size^33^. Hence, we hypothesized that the ES score would improve with at least 0.5 of baseline SD after four weeks of CBD-oil, estimated at four points based on earlier research^31^. To test this hypothesis with a paired sample t-test a sample size of 26 patients was required (one-sided alpha 5%, 80% power, estimated within-patient correlation 0.7). Also, differences in HR-QOL before and after CBD-oil use were determined and tested with a paired sample t-test or Wilcoxon signed rank test in case the scores were not normally distributed. Differences before and after CBD in specific side effects measured with the endocrine subscale of the FACT-ES were analyzed descriptively. The item ‘I am sleeping well’ is not part of the endocrine subscale but it is an item in the HR-QOL part. Because insomnia was frequently mentioned as a side effect of tamoxifen and CBD is known to potentially improve this, we also checked for changes in this item.

In an exploratory way, the difference in endoxifen AUC was analyzed for each CYP2D6 phenotype separately. If a difference between CYP2D6 phenotypes was found, the endoxifen C_min_ would be analyzed for different CYP2D6 phenotypes as well in all 26 patients.

### Supplementary information

Supplementary material

## Data Availability

All data supporting the findings of this study are available within the article and its information files and from the corresponding author upon reasonable request.
