# Supplementary material for: CBD-oil as a potential solution in case of severe tamoxifen-related side effects
Source: NPJ Breast Cancer. 2023 Aug 5;9:63. doi: 10.1038/s41523-023-00570-x (PMC10404290; doi:10.1038/s41523-023-00570-x)
Supplement: Supplementary file 1 — Supplementary material [file 41523_2023_570_MOESM1_ESM.pdf]

Supplementary information

Supplementary table 1. Tamoxifen pharmacokinetics with or without CBD per CYP2D6 phenotype

Pharmacokinetic parameters	Tamoxifen monotherapy[1]	Tamoxifen + CBD[1]	Relative difference (%) with vs without CBD (90% CI)
Intermediate metabolizers (IM)			
Tamoxifen $AUC_{0\text{-}24h}$ (N=8)[2]	8130(59)	7570 (64)	-6.9% (-13.2, -0.2%)
Tamoxifen C_{min} (N=13)[3]	321 (55)	309 (58)	-3.7% (-10.1, +3.1%)
Endoxifen $AUC_{0\text{-}24h}$ (N=8)[2]	623 (28)	494 (18)	-20.8% (-26.4, -14.8%)
Endoxifen C_{min} (N=13)[3]	26 (29)	22 (28)	-17.7% (-23.3, -11.7%)
Extensive metabolizers (EM)			
Tamoxifen $AUC_{0\text{-}24h}$ (N=7)[2]	6090 (76)	6210 (57)	+2.0% (-6.1, +10.8%)
Tamoxifen C_{min} (N=11)[3]	268 (59)	245 (51)	-8.5% (-18.6, +3.0%)
Endoxifen $AUC_{0\text{-}24h}$ (N=7)[2]	618 (15)	604 (13)	-2.2% (-11.1, +7.6%)
Endoxifen C_{min} (N=11)[3]	31 (46)	27 (34)	-13.7% (-21.9, -4.6%)

[1]geometric mean (coefficient of variation %) [2]all patients with IM and EM metabolism where AUC was determined [3]patients with IM and EM metabolism where AUC was determined AND all other patients with IM and EM metabolism where only C_{min} was determined

Supplementary table 2. FACT-ES items Endocrine subscale

Endocrine subscale items FACT-ES					
ES items	Not at all	A little bit	Some-what	Quite a bit	Very much
I have hot flashes	0	1	2	3	4
I have cold sweats	0	1	2	3	4
I have night sweats	0	1	2	3	4
I have vaginal discharge	0	1	2	3	4
I have vaginal itching/irritation	0	1	2	3	4
I have vaginal bleeding or spotting	0	1	2	3	4
I have vaginal dryness	0	1	2	3	4
I have pain or discomfort with intercourse	0	1	2	3	4
I have lost interest in sex	0	1	2	3	4
I have gained weight	0	1	2	3	4
I feel lightheaded/dizzy	0	1	2	3	4
I have been vomiting	0	1	2	3	4
I have diarrhea	0	1	2	3	4
I get headaches	0	1	2	3	4
I feel bloated	0	1	2	3	4
I have breast sensitivity/tenderness	0	1	2	3	4
I have mood swings	0	1	2	3	4
I am irritable	0	1	2	3	4
I have pain in my joints	0	1	2	3	4

Legend. *The FACT-ES has a 5 point liker-type response scale and contains 46 questions in total. It measures four domains of health-related quality of life: physical, social, emotional and functional well-being in 27 items. Also, it is a measure of side effects of endocrine treatments given in breast cancer patients (ES, 19 items, see above). Some items were negatively framed and were therefore reversed for analysis. High scores of the FACT-ES equate with good quality of life and/or experiencing few side effects while lower scores indicate poorer quality of life and/or experiencing many/severe side effects.*
